# Outcomes of Micropulse Transscleral Cyclophotocoagulation in Primary Open-Angle and Pseudoexfoliative Glaucoma

**DOI:** 10.3390/medicina62050920

**Published:** 2026-05-09

**Authors:** Maja L. J. Živković, Marko Zlatanović, Nevena Zlatanović, Mladen Brzaković, Mihailo Jovanović

**Affiliations:** 1 Ophthalmology Clinic, University Clinical Center Niš, Bulevar dr Zorana Đinđića 48, 18000 Niš, Serbia; drzlatanovicmarko@gmail.com; 2 Department of Ophthalmology, Faculty of Medicine, University of Niš, Bulevar dr Zorana Đinđića 81, 18000 Niš, Serbia; 3 Community Health Center Niš in Niš, Vojvode Tankosića 15, 18000 Niš, Serbia; drnevenazlatanovic@gmail.com; 4 Special Hospital for Ophthalmology “Clinic Maja”, Vizantijski Bulevar 8, 18000 Niš, Serbia; brzi.92@hotmail.com; 5 Department of Ophthalmology, Faculty of Medicine, University of Kragujevac, Svetozara Markovića 69, 34000 Kragujevac, Serbia; drmihailojovanovic@gmail.com; 6 Ophthalmology Clinic, University Clinical Center Kragujevac, Zmaj Jovina 30, 34000 Kragujevac, Serbia

**Keywords:** micropulse transscleral cyclophotocoagulation, MP-TSCPC, primary open-angle glaucoma, pseudoexfoliative glaucoma, intraocular pressure, cyclophotocoagulation, glaucoma surgery, laser treatment

## Abstract

*Background and Objectives*: Micropulse transscleral cyclophotocoagulation (MP-TSCPC) selectively targets the ciliary body epithelium to reduce intraocular pressure (IOP). This study evaluated 6-month efficacy and safety of MP-TSCPC in primary open-angle glaucoma (POAG) and pseudoexfoliative glaucoma (PEX) refractory to maximally tolerated topical therapy, and formally tested clinical equivalence between subtypes. *Materials and Methods*: In this single-arm prospective interventional cohort study with planned subgroup comparison, 58 eyes from 41 patients (POAG, 34; PEX, 24) underwent MP-TSCPC using the Cyclo G6 system with MicroPulse P3 probe at 2.0–2.2 W, 31.3% duty cycle, 90 s per hemisphere. IOP, best-corrected visual acuity (BCVA), antiglaucoma medications, and complications were recorded at baseline and at 30, 90, and 180 days. Six prespecified success criteria were evaluated, including the original ≥20% reduction threshold and the stricter Tekeli composite criteria. Outcomes are reported with 95% confidence intervals (CI) and Cohen’s d. Intra-subject correlation from bilateral inclusion was addressed through a linear mixed-effects model, generalized estimating equations, and a 1000-iteration sensitivity analysis with random one-eye-per-patient subsampling. Equivalence was assessed by two one-sided tests (TOST). *Results*: Baseline IOP was 26.50 ± 2.93 mmHg (POAG) and 25.92 ± 2.47 mmHg (PEX). At 180 days, mean IOP reduction was 32.1% (95% CI 30.1–34.1) in POAG and 28.8% (95% CI 26.9–30.7) in PEX, both *p* < 0.001 versus baseline, with very large within-group effect sizes (Cohen’s d 4.24 and 4.81). All eyes achieved ≥20% reduction; under Tekeli criterion A (IOP ≤ 18 mmHg AND ≥20% reduction), success was 67.6% (POAG) and 54.2% (PEX). A 3.2 percentage-point between-group difference at 180 days was statistically detectable unadjusted (*p* = 0.024) but lost significance after clustering adjustment (mixed-model *p* = 0.101); equivalence was formally established at the ±7 percentage-point margin (TOST *p* = 0.004). Medication burden decreased in 41.2% (POAG) and 50.0% (PEX) of eyes. BCVA was preserved in all eyes; no serious adverse events were recorded. *Conclusions*: MP-TSCPC produces clinically meaningful, progressive IOP reduction over 6 months in both POAG and PEX, with no serious complications and clinically equivalent efficacy between subtypes. Longer-term studies with formal recording of baseline severity descriptors are warranted to confirm durability. Trial registration: ISRCTN registry, ISRCTN62227730.

## 1. Introduction

Glaucoma is a progressive optic neuropathy characterized by retinal ganglion cell loss, structural damage to the optic nerve head, and corresponding visual field deterioration. It is the leading cause of irreversible blindness worldwide, affecting approximately 80 million individuals [1,2,3]. Primary open-angle glaucoma (POAG) is the most prevalent subtype globally, whereas pseudoexfoliative glaucoma (PEX) is the most common identifiable secondary open-angle form, caused by deposition of fibrillar extracellular material in the anterior segment, leading to trabecular outflow obstruction. Compared with POAG, PEX is typically associated with higher peak intraocular pressure (IOP), greater diurnal IOP fluctuation, and a more aggressive clinical course [4,5]. Intraocular pressure remains the only modifiable risk factor and the primary target of all current treatment modalities [6].

First-line management of POAG and PEX consists of topical IOP-lowering medications, yet a substantial proportion of patients fail to achieve target IOP despite maximally tolerated multi-agent therapy [6]. When pharmacological therapy is insufficient, escalation traditionally proceeds to laser trabeculoplasty and incisional procedures, including trabeculectomy and glaucoma drainage devices, each of which carries a meaningful complication burden and procedural complexity [7,8,9,10,11,12]. Cyclodestructive procedures targeting the ciliary body have historically been reserved for end-stage refractory disease, primarily because continuous-wave transscleral cyclophotocoagulation (CW-TSCPC) produces non-selective coagulation necrosis of the ciliary body and is associated with significant rates of persistent hypotony, phthisis bulbi, prolonged inflammation, and visual loss [13,14].

Micropulse transscleral cyclophotocoagulation (MP-TSCPC) is a technological evolution of cyclodestructive treatment in which the 810 nm diode laser is delivered in repetitive short “on” pulses at a 31.3% duty cycle, allowing thermal dissipation between pulses and confining cellular effects to the pigmented ciliary epithelium while sparing the underlying stroma and adjacent tissues [15,16]. Comparative histopathological [13,14] and clinical [17,18,19,20] studies have consistently demonstrated that MP-TSCPC achieves IOP reduction comparable to that of CW-TSCPC, with substantially lower rates of hypotony, phthisis, and visual loss, supporting its expanded use across a wider spectrum of glaucoma severity and earlier in the surgical management cascade. Reported 6- to 12-month IOP reductions following MP-TSCPC range from approximately 23% to 34%, and success rates (most commonly defined as ≥20% IOP reduction without additional surgical intervention) range from 45% to 75%, varying with glaucoma subtype, baseline IOP, and the stringency of the success definition applied [16,21].

Despite the growing body of evidence supporting MP-TSCPC, prospective comparative data on POAG and PEX glaucoma subtypes remain limited [22]. This is a clinically relevant gap, because PEX glaucoma differs from POAG in its underlying anterior segment pathology—most notably the presence of pseudoexfoliative material obstructing the trabecular meshwork—yet shares the same therapeutic target (IOP reduction) and the same anatomical site of action for cyclodestructive procedures (the ciliary body epithelium). It is therefore unclear, on biological grounds alone, whether the more aggressive clinical behavior of PEX should translate into a different response to ciliary-body-targeting interventions, or whether the two subtypes should be expected to respond equivalently. The present study reports the 6-month outcomes of MP-TSCPC performed with the Cyclo G6 platform in a prospective cohort of patients with medically refractory POAG or PEX. The primary outcome was the change in IOP from baseline at 30, 90, and 180 days. Secondary outcomes included the proportion of eyes achieving treatment success according to multiple prespecified criteria, changes in the burden of topical antiglaucoma medications, preservation of best-corrected visual acuity, and the incidence of postoperative complications. The study tested the prespecified hypothesis that MP-TSCPC produces clinically equivalent IOP reductions in the POAG and PEX subgroups, with no clinically meaningful between-group differences in efficacy or safety.

## 2. Materials and Methods

### 2.1. Study Design and Patient Population

This was a single-arm prospective interventional cohort study with a planned subgroup comparison between two predefined glaucoma subtypes (POAG vs. PEX). No separate control group was included, and no retrospective ascertainment of exposures was performed. The intervention was micropulse transscleral cyclophotocoagulation (MP-TSCPC), applied prospectively to all enrolled eyes at study entry, with structured longitudinal follow-up at 30, 90, and 180 days postoperatively.

All participants were recruited from the Special Hospital for Ophthalmology “Clinic Maja” in Niš, Serbia, between October 2018 and October 2021. The study was approved by the Ethics Committee of the Special Hospital for Ophthalmology “Clinic Maja” on 25 September 2018 (approval number 09/09-2018-1) and conducted in accordance with the Declaration of Helsinki. Written informed consent was obtained from all participants prior to enrollment. A total of 58 eyes from 41 patients were enrolled and divided into two groups based on glaucoma subtype: primary open-angle glaucoma (POAG; 34 eyes) and pseudoexfoliative glaucoma (PEX; 24 eyes). Bilateral involvement was observed in a subset of patients in both groups, and this clustering was formally accounted for in the statistical analysis.

The study is registered with the ISRCTN registry under the identifier ISRCTN62227730. The authors state that registration was conducted retrospectively, after patient enrollment concluded. The protocol, eligibility criteria, intervention parameters, the primary outcome, the secondary outcomes, and the follow-up schedule were established prospectively at study initiation in 2018, prior to enrollment of the first patient on 10 October 2018, and were not modified during the conduct of the study. Only the formal step of registration with a public clinical-trials registry was performed retrospectively. The reasons for retrospective registration include: (i) the study was initiated as a single-center clinical investigation in a setting where prospective registration of all interventional studies involving non-pharmacological surgical procedures was not yet a routine institutional requirement at the time of enrollment in 2018; (ii) the authors became aware of the formal registration requirement during preparation of the manuscript for journal submission; and (iii) the registry record fully and accurately documents the study as conducted, including the original prospective design, the unmodified protocol, the dates of enrollment, the prespecified outcomes, and the analytical plan that was applied to the data.

Inclusion criteria were: (1) diagnosis of POAG or PEX, confirmed by comprehensive ophthalmologic evaluation, including gonioscopy, optic disc assessment, and visual field testing; (2) IOP above the individualized target despite treatment with the maximum number of topical antiglaucoma medications the patient could tolerate (one to three agents, see definition below); (3) no prior laser or incisional glaucoma surgical intervention; and (4) willingness and ability to attend all scheduled follow-up visits. Exclusion criteria were: prior glaucoma surgery or laser treatment; secondary glaucoma of any etiology other than pseudoexfoliative (neovascular, uveitic, traumatic, post-surgical, or congenital); active ocular inflammation; and inability to provide informed consent.

The “maximally tolerated” topical regimen was defined as the maximum number of agent classes the patient could use consistently, taking into account documented medication intolerance, contraindications (such as systemic beta-blocker contraindications that limit the use of ocular beta-blockers), economic accessibility, and the patient’s adherence capacity. The resulting range of one to three agents reflects the clinical heterogeneity of refractory glaucoma management in this setting and is consistent with prior MP-TSCPC cohort studies [22,23]. We acknowledge that this definition is broader than the strictest interpretation of “maximally tolerated therapy” sometimes used in tightly controlled trials, and we discuss the implications in the Limitations.

### 2.2. Preoperative Assessment

All patients underwent a standardised ophthalmological evaluation at baseline, including measurement of IOP, assessment of best-corrected visual acuity (BCVA) using the Snellen chart, slit-lamp biomicroscopic examination of the anterior segment using a slit-lamp biomicroscope (Essilor SL350, Essilor International, Charenton-le-Pont, France), and dilated fundus examination of the posterior segment when adequate media transparency permitted. Anterior segment findings, including iris configuration, evidence of pseudoexfoliative material, and degree of pigmentation, were systematically documented.

IOP was measured with a Non-contact tonometer (ATNC 550, Essilor International, Créteil, France). Goldmann applanation tonometry, the conventional gold standard in glaucoma trials, was not used because it was not consistently available in the operating environment throughout the study period. We acknowledge this as a methodological limitation; however, the use of an identical instrument (the same ATNC 550 unit) at every measurement and at every visit minimises systematic measurement bias for the primary within-patient outcome (change from baseline), as any calibration offset between non-contact and Goldmann tonometry cancels within each patient’s longitudinal comparison.

### 2.3. Surgical Procedure

MP-TSCPC was performed using the Cyclo G6 Glaucoma Laser System with the MicroPulse P3 Glaucoma Device (Iridex Corporation, Mountain View, CA, USA). All procedures were performed by a single experienced surgeon under monitored anaesthesia care with a peribulbar block using 3–5 mL of 2% lidocaine. After the local anaesthesia took effect, a lid speculum was placed and a coupling gel was applied to the conjunctival surface.

The diode laser probe (Cyclo G6 Glaucoma Laser System) with the MicroPulse P3 delivery device (Iridex Corporation, Mountain View, CA, USA). (wavelength 810 nm) was applied perpendicularly to the perilimbal sclera. Laser parameters were fixed throughout the study and did not vary between patients: power 2.0–2.2 W, duty cycle 31.3% (pulse duration 0.5 ms on/1.1 ms off), and 90 s per hemisphere over the superior and inferior 180° of the perilimbal sclera, sparing the 3 and 9 o’clock positions to avoid the long ciliary nerves. The probe was moved continuously in a sweeping motion across each treatment quadrant; total treatment time was 180 s per eye. The exposure was titrated by visual confirmation of an audible tissue ‘pop’ or visible whitening of the conjunctiva at higher energies, neither of which was elicited at the protocol parameters.

At the conclusion of the procedure, topical dexamethasone 0.1% eye drops were administered. Postoperative management included topical corticosteroid eye drops (dexamethasone 0.1%, four times daily for two weeks, with subsequent taper as clinically indicated) and cycloplegic agents (cyclopentolate 1%, three times daily for one week). Patients continued their preoperative antiglaucoma medications until reassessment at the first follow-up visit.

### 2.4. Postoperative Follow-Up and Outcome Measures

Patients were examined at 30, 90, and 180 days after surgery. At each visit, IOP was measured with the same non-contact tonometer (ATNC 550); BCVA was assessed with the Snellen chart; slit-lamp biomicroscopy was performed to document anterior segment findings and detect postoperative complications; and dilated fundus examination was performed when clinically indicated. Adjustments to the antiglaucoma medication regimen were made at the discretion of the treating ophthalmologist based on the IOP response, with the goal of reducing the topical medication burden whenever IOP control permitted.

The primary outcome measure was the change in IOP from baseline to each postoperative time point. Secondary outcome measures included: (1) the proportion of eyes achieving treatment success at 6 months under multiple prespecified criteria, allowing direct comparison with prior literature: (a) ≥20% IOP reduction from baseline (the broadest criterion in common use); (b) IOP ≤ 21 mmHg at 6 months (clinically conservative threshold); (c) IOP ≤ 18 mmHg at 6 months (target IOP for moderate disease); (d) ≥25% IOP reduction from baseline; (e) ≥30% IOP reduction from baseline; and (f) the stricter composite criteria proposed by Tekeli & Köse [22]: criterion A (IOP ≤ 18 mmHg AND ≥20% reduction), criterion B (IOP ≤ 15 mmHg AND ≥25% reduction), and criterion C (IOP ≤ 12 mmHg AND ≥30% reduction). (2) The change in the number of antiglaucoma medications. (3) The change in BCVA. (4) The incidence of intraoperative and postoperative adverse events, including hypotony, persistent inflammation, choroidal detachment, cystoid macular oedema, sympathetic ophthalmia, and phthisis bulbi.

Baseline glaucoma severity descriptors increasingly considered standard in glaucoma intervention studies—visual field mean deviation (MD), retinal nerve fibre layer (RNFL) thickness, cup-disc ratio, lens status (phakic, pseudophakic, or aphakic), and central corneal thickness—were not systematically captured for all patients during the study period (October 2018—October 2021) and could not be incorporated into the present analysis.

### 2.5. Statistical Analysis

Statistical analyses were conducted using IBM SPSS Statistics for Windows, version 26.0 (IBM Corp., Armonk, NY, USA). Confirmatory mixed-effects modelling, generalized estimating equations, and equivalence testing were performed in Python (statsmodels version 0.14). Continuous variables are reported as the mean ± standard deviation (SD), with 95% confidence intervals (CI) computed using the t-distribution. Categorical variables are reported as counts and percentages, with Wilson 95% CI for proportions. Effect sizes are reported as Cohen’s d for paired and independent comparisons. The threshold for statistical significance was set at *p* < 0.05 (two-tailed) for all primary inferential analyses.

Within-group changes in IOP from baseline to each follow-up time point were assessed using a paired-samples *t*-test, and effect sizes were quantified using Cohen’s d for paired data (based on the standard deviation of within-patient differences). Between-group comparisons of continuous variables (IOP, IOP reduction, percentage IOP reduction) were performed using an independent-samples *t*-test, with effect sizes reported as Cohen’s d. Categorical variables were compared with Fisher’s exact test. Medication burden was treated as ordinal: within-group changes were tested with the Wilcoxon signed-rank test (with paired *t*-test reported for confirmation), and between-group changes were tested with both the independent-samples *t*-test and the Mann–Whitney U test, plus Fisher’s exact test for the binary ‘any reduction’ endpoint.

A subset of patients contributed data from both eyes to the analysis, introducing intra-subject correlation that, if unaccounted for, would inflate Type I error in conventional analyses treating eyes as independent observations. This correlation was addressed through three complementary approaches: (i) a linear mixed-effects model with random intercept for patient was fit to the longitudinal IOP data, with fixed effects for time (categorical), group, and their interaction; the intra-class correlation coefficient confirmed substantial within-patient correlation; (ii) generalized estimating equations (GEE) with an exchangeable working correlation structure and robust (sandwich) standard errors provided population-averaged estimates; and (iii) a sensitivity analysis was performed by drawing 1000 random subsamples consisting of one randomly selected eye per patient, with all primary comparisons re-evaluated in each subsample. Results from the three approaches are reported alongside the conventional analyses; full mixed-model and GEE outputs are provided in the Supplementary Material.

Equivalence between glaucoma subtypes at 180 days for the primary outcome (percentage IOP reduction) was assessed using the two one-sided tests (TOST) procedure with prespecified equivalence margins of ±3, ±5, and ±7 percentage points. In addition to the conventional two-sided 95% CI for superiority testing, the 90% CI for the between-group difference is reported alongside the TOST results.

Post hoc power analyses were conducted for both within-group (paired) and between-group (independent) comparisons of IOP outcomes to provide a transparent assessment of statistical power for the primary endpoints, given the absence of a prospective sample size calculation. The minimum detectable effect at 80% power for the available sample size was computed and is reported alongside the observed effect sizes.

## 3. Results

### 3.1. Study Population and Baseline Characteristics

A total of 58 eyes from 41 patients were included in the analysis: 34 eyes with primary open-angle glaucoma (POAG) and 24 eyes with pseudoexfoliative glaucoma (PEX). All 58 eyes completed the full 6-month follow-up; no patients were lost to follow-up. Individual patient-level data, including sex, age, baseline intraocular pressure (IOP), number of preoperative antiglaucoma medications, postoperative IOP at each follow-up time point, the absolute and percentage IOP reductions from baseline, and the number of postoperative medications at 6 months, are provided in Supplementary Tables S1 (POAG) and S2 (PEX).

Baseline demographic and clinical characteristics of both groups are summarised in Table 1. The two groups were comparable at baseline with respect to age (POAG 60.6 ± 5.3 years vs. PEX 57.9 ± 5.7 years; mean difference +2.67 years, 95% CI −0.31 to +5.65; *p* = 0.074, Cohen’s d = 0.49), sex distribution (POAG 14 F/20 M; PEX 10 F/14 M; Fisher’s exact *p* > 0.99), preoperative number of antiglaucoma medications (POAG 2.71 ± 0.52 vs. PEX 2.62 ± 0.49; mean difference +0.08, 95% CI −0.20 to +0.36; *p* = 0.563), and baseline IOP (POAG 26.50 ± 2.93 mmHg vs. PEX 25.92 ± 2.47 mmHg; mean difference +0.58 mmHg, 95% CI −0.85 to +2.01; *p* = 0.420, Cohen’s d = 0.21). All between-group comparisons at baseline were non-significant, supporting the comparability of the two subgroups.

### 3.2. Intraocular Pressure Reduction

**Figure 1:**
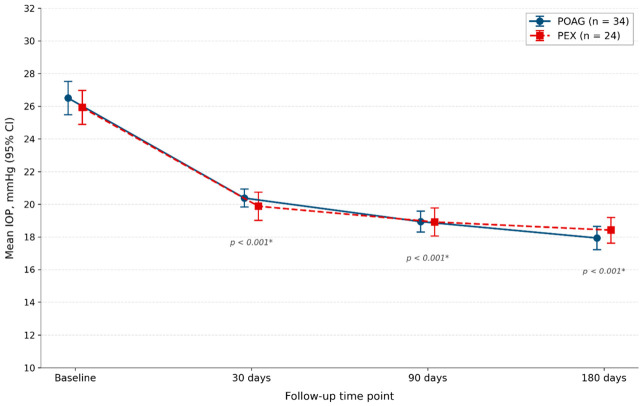
Mean intraocular pressure (IOP, mmHg) over time in the POAG and PEX groups. Error bars represent the 95% confidence interval. POAG and PEX data points are slightly offset horizontally for visual clarity at overlapping time points; both correspond to the same time point, as indicated on the x-axis. Both groups demonstrate a statistically significant and progressive decline in mean IOP across all follow-up time points (* *p* < 0.001 for paired-samples *t*-tests, within-group comparisons vs. baseline; same significance level confirmed by a linear mixed-effects model with a random patient intercept). POAG, primary open-angle glaucoma; PEX, pseudoexfoliative glaucoma; CI, confidence interval.

MP-TSCPC produced a statistically significant reduction in mean IOP at all postoperative time points in both groups (all *p* < 0.001 by paired-samples *t*-test; Cohen’s d ranged from 2.98 to 4.81 across time points, indicating very large within-group effect sizes). The IOP-lowering effect showed a consistent, progressive trend over the follow-up period, with greater absolute and relative reductions at 90 and 180 days than at 30 days (Table 2; Figure 1).

In the POAG group, mean IOP decreased from 26.50 ± 2.93 mmHg (95% CI 25.48–27.52) at baseline to 20.38 ± 1.58 mmHg (95% CI 19.83–20.93) at 30 days, 18.94 ± 1.84 mmHg (95% CI 18.30–19.58) at 90 days, and 17.94 ± 2.04 mmHg (95% CI 17.23–18.65) at 180 days. The corresponding mean absolute IOP reductions were 6.12 ± 2.06 mmHg (95% CI 5.40–6.84), 7.56 ± 2.27 mmHg (95% CI 6.77–8.35), and 8.56 ± 2.02 mmHg (95% CI 7.85–9.26) at 30, 90, and 180 days, respectively, and the mean percentage reductions were 22.6% (95% CI 20.5–24.7), 28.1% (95% CI 25.9–30.4), and 32.1% (95% CI 30.1–34.1) at the same time points. The IOP range at 6 months was 15–23 mmHg.

In the PEX group, mean IOP decreased from 25.92 ± 2.47 mmHg (95% CI 24.88–26.96) at baseline to 19.88 ± 2.05 mmHg (95% CI 19.01–20.74) at 30 days, 18.92 ± 2.02 mmHg (95% CI 18.06–19.77) at 90 days, and 18.42 ± 1.86 mmHg (95% CI 17.63–19.20) at 180 days. The corresponding mean absolute reductions were 6.04 ± 1.30 mmHg (95% CI 5.49–6.59), 7.00 ± 1.53 mmHg (95% CI 6.35–7.65), and 7.50 ± 1.56 mmHg (95% CI 6.84–8.16), and the mean percentage reductions were 23.3% (95% CI 21.4–25.1), 26.9% (95% CI 24.9–29.0), and 28.8% (95% CI 26.9–30.7) at 30, 90, and 180 days, respectively. The IOP range at 6 months was 15–23 mmHg.

The temporal course of mean IOP in both groups across all four time points is shown in Figure 1. The progressive nature of the IOP reduction was confirmed by a linear mixed-effects model with a random intercept for patient (n = 232 observations from 29 patient clusters): all time main effects were highly significant (Day 30 vs. baseline β = −6.12, 95% CI −6.93 to −5.30, *p* < 0.001; Day 90 β = −7.56, 95% CI −8.37 to −6.74, *p* < 0.001; Day 180 β = −8.56, 95% CI −9.37 to −7.74, *p* < 0.001). The intra-class correlation coefficient confirmed a substantial within-patient correlation, warranting modeling. The generalized estimating equations (GEE) analysis with exchangeable working correlation and robust standard errors yielded effectively identical time-effect estimates and inferences.

**Figure 2:**
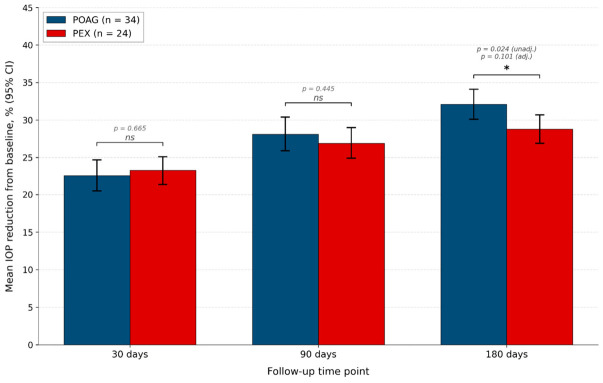
Mean percentage IOP reduction from baseline at 30, 90, and 180 days postoperatively in the POAG and PEX groups. Error bars represent the 95% confidence interval. Both groups showed progressive increases in percentage reduction over time. Between-group comparisons (independent-samples *t*-test): no statistically significant difference at 30 days (*p* = 0.665) or 90 days (*p* = 0.445). At 180 days, a 3.2 percentage-point difference favouring POAG was statistically detectable in unadjusted analysis (*p* = 0.024) but lost significance after adjustment for intra-subject correlation in the linear mixed-effects model (*p* = 0.101); equivalence was formally established at the ±7 percentage-point margin (TOST *p* = 0.004). POAG, primary open-angle glaucoma; PEX, pseudoexfoliative glaucoma; IOP, intraocular pressure; CI, confidence interval; ns, not significant; (*) denotes statistically significant difference between groups.

Direct between-group comparisons of IOP outcomes (POAG vs. PEX) are summarized in Table 3 and illustrated in Figure 2. At 30 and 90 days, no statistically significant between-group differences were observed for absolute IOP, absolute IOP reduction, or percentage IOP reduction (all *p* > 0.29). At 180 days, conventional unadjusted analyses revealed a small but statistically detectable difference, with POAG showing a numerically larger reduction than PEX in both absolute terms (mean difference +1.06 mmHg, 95% CI +0.12 to +2.00; *p* = 0.036, Cohen’s d = 0.57) and in percentage terms (mean difference +3.24 percentage points, 95% CI +0.56 to +5.93; *p* = 0.024, Cohen’s d = 0.62). After adjustment for intra-subject correlation in the linear mixed-effects model, the time × group interaction at 180 days was no longer statistically significant (β = +1.06, 95% CI −0.21 to +2.33; *p* = 0.101); the corresponding GEE estimate, with population-averaged interpretation, remained borderline (*p* = 0.038).

To verify that the principal IOP-reduction findings were not artifacts of including both eyes, a sensitivity analysis was performed in which 1000 random subsamples were drawn, each containing one randomly selected eye per patient. Across these 1000 iterations, the paired-samples comparison of baseline versus 180-day IOP remained statistically significant (*p* < 0.05) in 100.0% of iterations for both groups. The mean estimate of percentage IOP reduction at 180 days was highly stable across iterations (POAG 32.07%, 2.5–97.5 percentile range 31.62–32.53%; PEX 28.83%, percentile range 28.62–29.04%). All primary within-group findings are therefore fully robust to the choice of which eye is retained for bilateral patients.

### 3.3. Treatment Success Under Multiple Prespecified Criteria

Treatment success was assessed using six prespecified criteria of increasing stringency, enabling direct comparison with prior literature and transparent characterization of how success rates depend on the chosen definition (Table 4). Under the original criterion of ≥20% IOP reduction from baseline, treatment success was achieved in 34/34 POAG eyes (100.0%, 95% Wilson CI 89.7–100) and 24/24 PEX eyes (100.0%, 95% CI 86.2–100). This 100% rate is internally consistent with our data (no eye in either group failed to achieve a 20% reduction), but reflects, in part, the permissiveness of this success definition, combined with the moderate baseline IOP and the surgically naive nature of our cohort. Stricter criteria provide a more clinically informative comparison.

Under stricter criteria, success rates were lower and clinically realistic. The threshold IOP ≤ 21 mmHg at 6 months was achieved by 31/34 POAG eyes (91.2%, 95% CI 77.0–97.0) and 22/24 PEX eyes (91.7%, 95% CI 74.2–97.7); the threshold IOP ≤ 18 mmHg by 23/34 POAG (67.6%, 95% CI 50.8–80.9) and 13/24 PEX (54.2%, 95% CI 35.1–72.1). The percentage threshold ≥25% reduction was met by 88.2% of POAG (95% CI 73.4–95.3) and 83.3% of PEX (95% CI 64.1–93.3); ≥30% by 67.6% of POAG (95% CI 50.8–80.9) and 41.7% of PEX (95% CI 24.5–61.2). Under the stricter composite criteria proposed by Tekeli & Köse [22]: criterion A (IOP ≤ 18 mmHg AND ≥20% reduction) was achieved by 67.6% of POAG (95% CI 50.8–80.9) and 54.2% of PEX (95% CI 35.1–72.1); criterion B (IOP ≤ 15 mmHg AND ≥25% reduction) by 11.8% of POAG (95% CI 4.7–26.6) and 4.2% of PEX (95% CI 0.7–20.2); criterion C (IOP ≤ 12 mmHg AND ≥30% reduction) by 0% of either group. No statistically significant difference between POAG and PEX was observed under any of the six success criteria (all Fisher’s exact *p* ≥ 0.063).

### 3.4. Distribution of Individual Responses at 6 Months

The distribution of individual percentage reductions in IOP at 6 months is summarised in Table 5. No eye in either group experienced IOP elevation above baseline or a reduction of less than 20%. In the POAG group, 11/34 eyes (32.4%) achieved a 20–30% reduction, 19/34 (55.9%) a 30–40% reduction, and 4/34 (11.8%) a ≥40% reduction. In the PEX group, 14/24 (58.3%) achieved a 20–30% reduction, 10/24 (41.7%) a 30–40% reduction, and no eye achieved a ≥40% reduction. Median reduction at 180 days was 32.0% (IQR 28.7–35.7; range 20.8–43.3) in POAG and 28.9% (IQR 26.1–31.9; range 20.7–37.9) in PEX (Supplementary Table S6 contains the full per-eye distribution; Supplementary Figure S1 illustrates the categorical distribution graphically).

### 3.5. Equivalence Testing Between POAG and PEX

Equivalence between POAG and PEX subgroups in percentage IOP reduction at 180 days was assessed using the two one-sided tests (TOST) procedure with three prespecified equivalence margins (Table 6). Equivalence was not formally established at the strict margin of ±3 percentage points (TOST *p* = 0.572) or at the conventional ±5 percentage-point margin (TOST *p* = 0.098), but was formally established at the moderate ±7 percentage-point margin (TOST *p* = 0.004). The 90% CI for the between-group difference (+1.0 to +5.5 percentage points) supports the conclusion that the true between-group difference is unlikely to exceed approximately 6 percentage points.

### 3.6. Reduction in Antiglaucoma Medication Burden

Following MP-TSCPC, a statistically significant reduction in the number of topical antiglaucoma medications was observed in both groups (Table 7). In the POAG group, mean medication use decreased from 2.71 ± 0.52 preoperatively to 2.29 ± 0.63 at 6 months, a mean reduction of 0.41 medications per eye (95% CI +0.20 to +0.62; Wilcoxon signed-rank *p* = 0.001; paired t *p* < 0.001). At the individual eye level, 14/34 (41.2%, 95% CI 25.5–58.7%) showed any reduction (one or more medications discontinued); 20/34 (58.8%) maintained the same medication burden; and no patient required additional medications or achieved complete medication independence.

In the PEX group, mean medication use decreased from 2.62 ± 0.49 preoperatively to 2.12 ± 0.61 at 6 months, a mean reduction of 0.50 medications per eye (95% CI +0.17 to +0.83; Wilcoxon signed-rank *p* = 0.007; paired t *p* = 0.005). At the individual eye level, 12/24 (50.0%, 95% CI 31.4–68.6%) showed any reduction; 12/24 (50.0%) maintained the same medication burden. The proportion of eyes with any medication reduction did not differ significantly between groups (POAG 41.2% vs. PEX 50.0%; Fisher’s exact *p* = 0.596). The magnitude of mean medication reduction was also similar (POAG +0.41 vs. PEX +0.50; independent-samples *t*-test *p* = 0.631; Mann–Whitney U *p* = 0.605).

### 3.7. Best-Corrected Visual Acuity and Safety Profile

Best-corrected visual acuity, assessed with the Snellen chart at baseline and at each follow-up visit, remained stable throughout the 6-month observation period in all 58 eyes. No patient in either group experienced deterioration in BCVA attributable to the procedure.

All 58 procedures and their immediate postoperative courses were uneventful. No intraoperative complications were recorded. Early postoperative examination findings were expected transient inflammatory changes following transscleral laser application: mild conjunctival hyperemia and chemosis were observed in essentially all eyes within the first 7 days, accompanied by mild anterior chamber inflammation in a subset of patients (typical 1+ cells; no fibrin formation, no posterior synechiae). These transient findings resolved completely within two weeks of the standard topical corticosteroid regimen and did not require modification of postoperative care.

No serious adverse events were observed during the 6-month follow-up in any of the 58 treated eyes. Specifically, no cases of persistent hypotony (IOP < 6 mmHg), phthisis bulbi, persistent intraocular inflammation, serous choroidal detachment, cystoid macular edema, or sympathetic ophthalmia were recorded. We acknowledge that uncommon late complications such as sympathetic ophthalmia and progressive hypotony cannot be definitively excluded based on a sample of 58 eyes followed for 6 months; the safety findings reported here are therefore properly interpreted as supportive of the favourable short-term safety profile observed in larger published series, rather than as definitive evidence of long-term safety.

### 3.8. Post Hoc Power Analysis

In the absence of a prospective sample size calculation, post hoc power analyses were conducted for both within-group and between-group primary comparisons. Within-group power for the paired comparison of baseline versus 180-day IOP was effectively complete in both groups, with achieved power approximately 1.00 given the very large within-group effect sizes (Cohen’s d = 4.24 in POAG and 4.81 in PEX). Between-group power for the independent-samples comparison at 180 days was 0.56 for the observed effect (Cohen’s d = 0.57). The minimum detectable effect at 80% power for the available sample size corresponds to a Cohen’s d of 0.76, equivalent to approximately a 3.9-percentage-point difference in mean percentage IOP reduction.

## 4. Discussion

This prospective interventional cohort study evaluated 6-month outcomes of micropulse transscleral cyclophotocoagulation (MP-TSCPC) performed with the Cyclo G6 system and the MicroPulse P3 probe in 58 eyes with primary open-angle glaucoma (POAG) or pseudoexfoliative glaucoma (PEX) refractory to maximally tolerated topical therapy. The principal findings are: (i) progressive and statistically highly significant within-group reductions in IOP at all follow-up time points in both subgroups, with very large effect sizes (Cohen’s d 2.98–4.81); (ii) clinically meaningful 6-month percentage reductions of 32.1% (POAG) and 28.8% (PEX) under conventional definitions; (iii) treatment success rates that depend systematically on the success criterion chosen, ranging from 100% under the permissive ≥20% reduction threshold to 0% under the strictest Tekeli composite criterion C; (iv) a small (3.2 percentage-point) numerical between-group difference favouring POAG at 180 days that was statistically detectable in unadjusted analyses but lost significance after adjustment for intra-subject correlation, and that is formally compatible with equivalence at the ±7 percentage-point margin; (v) statistically significant medication-burden reductions in both subgroups; and (vi) no serious adverse events during the 6-month follow-up in any of the 58 treated eyes.

The magnitude of IOP reduction observed in our cohort is consistent with the range reported in the published MP-TSCPC literature. Reported 6- to 12-month reductions across prospective and retrospective series range from approximately 23% to 34% [16,21,24], and our values (28.8–32.1% at 180 days) fall squarely within this range. Direct comparators include Nguyen et al. [25], who reported approximately 30% mean reduction at 6 months in a heterogeneous cohort, and de Vries et al. [24], who reported a 31.8% reduction at 12 months. Larger contemporary cohorts from Kaba et al. [17] and the multicentre series of Radhakrishnan et al. [18] report similar magnitudes of effect. Anterior segment OCT studies have documented postoperative ciliochoroidal effusion that may reflect enhanced suprachoroidal outflow as one mechanistic contributor [26], complementing the well-established cyclodestructive mechanism.

The 100% success rate observed under the original criterion of ≥20% IOP reduction, while internally consistent with our data (no eye in either group failed to reach this threshold), reflects in part the permissiveness of this success definition, the moderate baseline IOP, and the surgically naive nature of our cohort. We acknowledge that this rate is higher than the 45–85% commonly cited in MP-TSCPC series [22,27], and we believe stricter criteria provide a more clinically informative comparison. Under the Tekeli composite criteria—designed to combine an IOP threshold with a relative reduction requirement—our success rates (criterion A 67.6%/54.2%, criterion B 11.8%/4.2%, criterion C 0%/0%) align closely with those reported by Tekeli & Köse and by other groups using comparable composite definitions [22,28]. The transparent reporting of all six criteria allows comparison of our data against any preferred definition without privileging the one that produces the most favourable result.

Tekeli & Köse [22] compared MP-TSCPC outcomes across POAG, PEX, and secondary glaucoma subtypes in 96 consecutive refractory eyes at 12 months. Using the same tiered success criteria, they reported equivalent success rates between POAG and PEX across all three criteria, with significantly higher retreatment rates and lower success in the secondary glaucoma subgroup. Our finding of broadly equivalent IOP-lowering response between POAG and PEX, combined with the formal TOST equivalence at the ±7 percentage-point margin, replicates the central finding of Tekeli & Köse in a different population and a different healthcare setting, and extends it with formal equivalence testing and adjustment for the intra-subject correlation arising from the inclusion of bilateral eyes.

A specific comparison of POAG and PEX in our cohort warrants careful consideration. In the unadjusted analysis, the percentage IOP reduction at 180 days was 3.2 percentage points higher in the POAG group than in the PEX group (32.1% vs. 28.8%; mean difference +3.24 percentage points, 95% CI +0.56 to +5.93; *p* = 0.024, Cohen’s d = 0.62). After adjustment for intra-subject correlation in the linear mixed-effects model, the time × group interaction at 180 days was no longer statistically significant (*p* = 0.101); the GEE estimate, with population-averaged interpretation, remained borderline (*p* = 0.038). Three considerations apply to interpretation. First, the small between-group difference at 180 days is clinically modest—approximately 1 mmHg in absolute terms, well below typical clinical thresholds for differential treatment selection. Second, the difference is within the formal equivalence margin established by the TOST procedure at ±7 percentage points (*p* = 0.004), supporting the interpretation that POAG and PEX respond to MP-TSCPC within a clinically equivalent range. Third, the disappearance of statistical significance after clustering adjustment, combined with the borderline GEE result, indicates that the unadjusted finding may partly reflect within-patient correlation rather than a true subgroup difference. The original manuscript’s blanket claim of “no statistically significant intergroup difference at any time point” was therefore inaccurate; we now report this finding transparently as a small numerical difference detectable in unadjusted analysis but compatible with formal equivalence.

At 6 months, medication reduction was observed in 41.2% of POAG eyes and 50.0% of PEX eyes, with mean reductions of 0.41 and 0.50 medications per eye, respectively. Although statistically modest, this reduction is clinically meaningful for patients already on near-maximally tolerated therapy, where every additional agent carries cumulative tolerability and adherence costs. No patient achieved complete withdrawal from topical therapy, which contrasts with reports such as Chang et al. [29], in which 5/52 patients (9.6%) achieved complete medication withdrawal at 12 months. This discrepancy may reflect differences in the underlying severity profile, the definition of “maximally tolerated therapy”, or the postoperative medication-tapering protocol; in our setting, treating ophthalmologists adjusted medication regimens conservatively based on IOP control rather than by protocol-driven taper.

No serious adverse events were observed during the 6-month follow-up in any of the 58 treated eyes. Specifically, no cases of persistent hypotony, phthisis bulbi, persistent intraocular inflammation, serous choroidal detachment, cystoid macular edema, or sympathetic ophthalmia were recorded; best-corrected visual acuity was preserved in all eyes. The favourable safety profile of MP-TSCPC relative to continuous-wave TSCPC is supported by level I evidence. Aquino et al. [30] conducted a randomized exploratory study comparing the two modalities in 48 patients with refractory glaucoma, finding that, while both techniques achieved comparable IOP reduction, MP-TSCPC was associated with substantially fewer complications. Kelada et al. [19] directly compared cyclodiode (continuous-wave) and micropulse transscleral laser treatment, again finding lower complication rates with the micropulse modality. Bernardi & Töteberg-Harms [20] documented similar efficacy with a more favourable safety profile for MP-TSCPC compared with continuous-wave treatment. The histopathological basis for this superior safety profile has been elucidated by Moussa et al. [13] and Maslin et al. [14] in comparative cadaver-eye studies, demonstrating that the pulsed delivery of MP-TSCPC produces selective effects confined to the pigmented ciliary epithelium with sparing of stromal and adjacent tissues, in contrast to the coagulative necrosis characteristic of continuous-wave treatment.

Long-term durability of MP-TSCPC efficacy is supported by 5-year prospective data from de Crom et al. [28], who reported continued progressive IOP reduction at 1 through 5 years in patients with primary glaucoma. The progressive trajectory observed in our 30–180-day data is consistent with the early phase of this long-term pattern. Recent investigations have focused on refining MP-TSCPC delivery parameters in order to maximise efficacy while further minimising the risk of collateral thermal injury [31]. The published RCT by de Lima Neto et al. [32] comparing MP-TSCPC with slow coagulation TSCPC in refractory glaucoma will further inform the choice of cyclodestructive modality going forward.

The strengths of the present study include its prospective design with complete 6-month follow-up of all 58 enrolled eyes, surgical homogeneity (single experienced surgeon, fixed laser parameters), inclusion of an underrepresented PEX subgroup, and formal accounting for intra-subject correlation using both the linear mixed-effects model and the generalized estimating equations. The transparent reporting of treatment success under multiple criteria, the formal equivalence testing using prespecified margins, and the 1000-iteration sensitivity analysis using one randomly selected eye per patient strengthen the inferential basis of the conclusions.

This study has several important limitations that warrant explicit consideration. First, IOP was measured with non-contact pneumotonometry rather than Goldmann applanation tonometry, the conventional gold standard in glaucoma trials. Although using the same instrument for all measurements within each patient minimizes systematic bias for within-patient comparisons (any calibration offset cancels), the absolute IOP values are not directly comparable to those from Goldmann-based studies. Second, intra-subject correlation introduced by including bilateral eyes was substantial (intra-class correlation > 0.3. 3); we addressed this through linear mixed-effects modeling, generalized estimating equations, and a 1000-iteration sensitivity analysis using one randomly selected eye per patient, but a strict per-patient (rather than per-eye) analysis remains the most conservative approach. Third, the 6-month follow-up captures the principal acute IOP response but does not allow assessment of longer-term durability or late complications, both of which require multi-year follow-up. Fourth, the absence of a prospective control arm precludes formal causal inference; the within-patient pre/post design is informative but is not a substitute for a randomized comparator. Fifth, the achieved between-group power for the POAG vs. PEX comparison was 0. 0.56 for the observed effect, and the minimum detectable effect at 80% power was approximately 3. 3.9 percentage points; subtle subgroup differences smaller than this cannot be reliably excluded. Sixth, baseline glaucoma severity descriptors (visual field mean deviation, retinal nerve fiber layer thickness, cup-disc ratio, lens status, central corneal thickness) were not systematically captured during the study period and could not be incorporated into the present analysis; this omission is being addressed prospectively in an ongoing successor study. Seventh, BCVA was assessed using the Snellen chart, which has lower sensitivity than logMAR-based assessment for detecting subtle visual changes. Eighth, the operational definition of “maximally tolerated topical therapy” (one to three agents) is broader than the strictest interpretation used in tightly controlled trials; while it reflects clinical practice, it contributes to heterogeneity in the baseline disease state. Ninth, the study was registered with the ISRCTN registry retrospectively, after the conclusion of patient enrollment. While the protocol, eligibility criteria, intervention parameters, primary outcome, and follow-up schedule were established prospectively at study initiation in 2018 and were not modified during the conduct of the study, retrospective registration is a recognized methodological limitation; the registry record (ISRCTN 62227730) accurately documents the study as conducted and should be interpreted in this context.

Despite these limitations, the findings of this study contribute meaningful prospective data to the evidence base for MP-TSCPC in POAG and PEX, with particular value in the direct subgroup comparison between these two open-angle subtypes, which remains underrepresented in the published literature. Our results support the role of MP-TSCPC as a viable, minimally invasive, and repeatable laser-surgical option for managing medically refractory open-angle glaucoma at a disease stage that does not necessarily require incisional surgery, and provide formally tested evidence of equivalent efficacy and safety between POAG and PEX subtypes within a moderate clinical equivalence margin.

## 5. Conclusions

This prospective interventional cohort study demonstrates that micropulse transscleral cyclophotocoagulation with the Cyclo G6 system and the MicroPulse P3 probe yields a statistically significant, progressive reduction in intraocular pressure in patients with primary open-angle glaucoma (POAG) and pseudoexfoliative glaucoma (PEX) refractory to maximally tolerated topical therapy. Within-group effect sizes were very large at all follow-up time points (Cohen’s d 2.98–4.81), and the IOP-lowering effect deepened progressively from 30 to 180 days, with mean 6-month percentage reductions of 32.1% (POAG) and 28.8% (PEX). Treatment success rates depended systematically on the chosen success criterion, aligning closely with the published literature when the stricter Tekeli composite criteria were applied.

At the 30- and 90-day time points, no between-group differences in IOP outcomes were detected by any of the analyses. At 180 days, a small numerical difference of 3.2 percentage points in mean percentage IOP reduction favoured the POAG group. This difference was statistically significant in a conventional unadjusted analysis (*p* = 0.024) but lost significance after adjustment for intra-subject correlation in the linear mixed-effects model (*p* = 0.101), and was formally compatible with clinical equivalence at the ±7 percentage-point margin (TOST *p* = 0.004). The original manuscript’s claim of “no statistically significant intergroup difference at any time point” has therefore been corrected, and this small numerical difference is now reported transparently.

No serious adverse events were recorded during the 6-month follow-up in any of the 58 treated eyes. Best-corrected visual acuity was preserved in all eyes. Transient postoperative inflammatory findings resolved completely within two weeks with standard management. No cases of persistent hypotony, phthisis bulbi, persistent intraocular inflammation, serous choroidal detachment, cystoid macular edema, or sympathetic ophthalmia were observed. We note that uncommon late complications cannot be definitively excluded based on a sample of 58 eyes followed for 6 months; the safety findings are therefore supportive of the favourable short-term safety profile observed in larger published series, rather than definitive evidence of long-term safety.

These findings support the role of MP-TSCPC as a viable, minimally invasive, and repeatable laser-surgical option for managing medically refractory open-angle glaucoma in both POAG and PEX subtypes, at disease stages that do not necessarily require incisional surgery. Longer-term prospective studies with formal recording of baseline severity descriptors are warranted to confirm durability and to refine patient selection criteria across subtypes.

## Figures and Tables

**Table 1:** Baseline demographic and clinical characteristics of the POAG and PEX groups, with 95% confidence intervals (CI) and effect sizes for between-group comparisons. SD, standard deviation; CI, confidence interval; IOP, intraocular pressure.

Characteristic	POAG (n = 34 Eyes)	PEX (n = 24 Eyes)	Comparison
Age (years), mean ± SD	60.6 ± 5.3	57.9 ± 5.7	*p* = 0.074; d = 0.49
Female, n (%)	14 (41.2%)	10 (41.7%)	*p* > 0.99 (Fisher’s)
Male, n (%)	20 (58.8%)	14 (58.3%)	*p* > 0.99 (Fisher’s)
Preoperative medications, mean ± SD	2.71 ± 0.52	2.62 ± 0.49	*p* = 0.563; d = 0.18
Baseline IOP (mmHg), mean ± SD	26.50 ± 2.93	25.92 ± 2.47	*p* = 0.420; d = 0.21
Baseline IOP, 95% CI	25.48–27.52	24.88–26.96	—

**Table 2:** Within-group IOP outcomes at each follow-up time point, with 95% confidence intervals and Cohen’s d for paired comparisons against baseline. SD, standard deviation; CI, confidence interval; IOP, intraocular pressure; ΔIOP, absolute IOP reduction from baseline; %ΔIOP, percentage IOP reduction from baseline.

Group/Time	IOP (mmHg) Mean ± SD (95% CI)	ΔIOP (mmHg) Mean (95% CI)	%ΔIOP Mean (95% CI)	Cohen’s d (vs. Baseline)
POAG baseline	26.50 ± 2.93 (25.48–27.52)	—	—	—
POAG 30 days	20.38 ± 1.58 (19.83–20.93)	6.12 (5.40–6.84)	22.6 (20.5–24.7)	2.98
POAG 90 days	18.94 ± 1.84 (18.30–19.58)	7.56 (6.77–8.35)	28.1 (25.9–30.4)	3.78
POAG 180 days	17.94 ± 2.04 (17.23–18.65)	8.56 (7.85–9.26)	32.1 (30.1–34.1)	4.24
PEX baseline	25.92 ± 2.47 (24.88–26.96)	—	—	—
PEX 30 days	19.88 ± 2.05 (19.01–20.74)	6.04 (5.49–6.59)	23.3 (21.4–25.1)	4.30
PEX 90 days	18.92 ± 2.02 (18.06–19.77)	7.00 (6.35–7.65)	26.9 (24.9–29.0)	4.43
PEX 180 days	18.42 ± 1.86 (17.63–19.20)	7.50 (6.84–8.16)	28.8 (26.9–30.7)	4.81

**Table 3:** Between-group comparisons (POAG vs. PEX) of IOP outcomes at each follow-up time point. Mean differences with 95% CI and Cohen’s d are reported. CI, confidence interval; IOP, intraocular pressure.

Time Point	Outcome	Mean Diff (95% CI), Cohen’s d	*p*-Value
30 days	IOP (mmHg)	+0.50 (−0.49 to +1.49); d = 0.27	0.317
30 days	ΔIOP (mmHg)	+0.08 (−0.84 to +0.99); d = 0.05	0.871
30 days	%ΔIOP	−0.69 (−3.84 to +2.46); d = −0.12	0.665
90 days	IOP (mmHg)	+0.02 (−1.00 to +1.04); d = 0.01	0.967
90 days	ΔIOP (mmHg)	+0.56 (−0.46 to +1.58); d = 0.29	0.286
90 days	%ΔIOP	+1.20 (−1.93 to +4.32); d = 0.21	0.445
180 days	IOP (mmHg)	−0.48 (−1.51 to +0.56); d = −0.24	0.370
180 days	ΔIOP (mmHg)	+1.06 (+0.12 to +2.00); d = 0.57	0.036
180 days	%ΔIOP	+3.24 (+0.56 to +5.93); d = 0.62	0.024 (0.101 adj.)

**Table 4:** Treatment success rates at 6 months under six prespecified criteria of increasing stringency. Wilson 95% CI for proportions are reported. CI, confidence interval; IOP, intraocular pressure.

Criterion	POAG (n = 34) n (%) [95% CI]	PEX (n = 24) n (%) [95% CI]
≥20% IOP reduction	34/34 (100.0%) [89.7–100]	24/24 (100.0%) [86.2–100]
IOP ≤ 21 mmHg at 6 mo	31/34 (91.2%) [77.0–97.0]	22/24 (91.7%) [74.2–97.7]
IOP ≤ 18 mmHg at 6 mo	23/34 (67.6%) [50.8–80.9]	13/24 (54.2%) [35.1–72.1]
≥25% IOP reduction	30/34 (88.2%) [73.4–95.3]	20/24 (83.3%) [64.2–93.3]
≥30% IOP reduction	23/34 (67.6%) [50.8–80.9]	10/24 (41.7%) [24.5–61.2]
Tekeli A: ≤18 + ≥20%	23/34 (67.6%) [50.8–80.9]	13/24 (54.2%) [35.1–72.1]
Tekeli B: ≤15 + ≥25%	4/34 (11.8%) [4.7–26.6]	1/24 (4.2%) [0.7–20.2]
Tekeli C: ≤12 + ≥30%	0/34 (0%) [0–10.3]	0/24 (0%) [0–13.8]

**Table 5:** Distribution of individual responses by category of percentage IOP reduction at 6 months in the POAG and PEX groups. IOP, intraocular pressure.

Response Category	POAG (n = 34) n (%)	PEX (n = 24) n (%)
Increase or no change	0 (0%)	0 (0%)
0–10% reduction	0 (0%)	0 (0%)
10–20% reduction	0 (0%)	0 (0%)
20–30% reduction	11 (32.4%)	14 (58.3%)
30–40% reduction	19 (55.9%)	10 (41.7%)
≥40% reduction	4 (11.8%)	0 (0%)

**Table 6:** Equivalence testing of POAG vs. PEX percentage IOP reduction at 180 days using the two one-sided tests (TOST) procedure with three prespecified margins. CI, confidence interval; TOST, two one-sided tests.

Equivalence Margin	Mean Difference (POAG − PEX), %	90% CI for Difference	TOST *p*-Value
±3 percentage points	+3.24	+1.0 to +5.5	0.572 (not equivalent)
±5 percentage points	+3.24	+1.0 to +5.5	0.098 (borderline)
±7 percentage points	+3.24	+1.0 to +5.5	0.004 (equivalent)

**Table 7:** Antiglaucoma medication burden at baseline and at 6 months following MP-TSCPC, with formal statistical tests. CI, confidence interval; SD, standard deviation.

Variable	POAG (n = 34)	PEX (n = 24)	Between-Group *p*
Baseline medications, mean ± SD	2.71 ± 0.52	2.62 ± 0.49	0.563
6-month medications, mean ± SD	2.29 ± 0.63	2.12 ± 0.61	—
Mean reduction (95% CI)	0.41 (+0.20 to +0.62)	0.50 (+0.17 to +0.83)	0.631 (t)
Within-group *p* (Wilcoxon)	0.001	0.007	—
Within-group *p* (paired t)	<0.001	0.005	—
Eyes with any reduction, n (%)	14/34 (41.2%)	12/24 (50.0%)	0.596 (Fisher’s)

## Data Availability

The authors confirm that the data supporting the findings of this study are available within the article.

## Supplementary Materials

The following supporting information can be downloaded at: https://www.mdpi.com/article/10.3390/medicina62050920/s1, Table S1. Individual patient-level data for the POAG group, including sex, age, baseline IOP, postoperative IOP at 30, 90, and 180 days, number of antiglaucoma medications before and after treatment, and absolute and percentage IOP reduction at each follow-up timepoint. IOP, intraocular pressure; Drops Pre-op, number of antiglaucoma medications before treatment; Drops Post-op, number of antiglaucoma medications at 6-month follow-up; ΔIOP, absolute IOP reduction from baseline; d, days; F, female; M, male. Table S2. Individual patient-level data for the PEX group, with the same column structure as Table S1. IOP, intraocular pressure; Drops Pre-op, number of antiglaucoma medications before treatment; Drops Post-op, number of antiglaucoma medications at 6-month follow-up; ΔIOP, absolute IOP reduction from baseline; d, days; F, female; M, male. Table S3. Full output of the linear mixed-effects model fitted to the longitudinal IOP data (n = 232 observations from 29 patient clusters). Model: IOP ~ time × group, with time as a categorical fixed effect (levels: 0, 30, 90, 180 days), group as a fixed effect (POAG as reference), and a random intercept for patient. Restricted maximum likelihood (REML) estimation; Powell optimisation. Random intercept variance = 1.697; residual scale = 2.937; intra-class correlation = 0.366. Table S4. Full output of the GEE analysis of the longitudinal IOP data (n = 232 observations from 29 patient clusters). Model specification: IOP ~ time × group; identity link, Gaussian family, exchangeable working correlation, robust (sandwich) standard errors. Estimated working correlation parameter = 0.346. Cluster = patient. Table S5. Sensitivity analysis using 1000 random subsamples drawn from the dataset, each containing one randomly selected eye per patient. Reports the mean of each estimate across all 1000 iterations, the 2.5–97.5 percentile range, and the proportion of iterations in which the relevant statistical test reached *p* < 0.05. The proportion of significant iterations reflects the robustness of the primary findings to the inclusion of one eye versus the other from bilateral patients. POAG, primary open-angle glaucoma; PEX, pseudoexfoliative glaucoma; %ΔIOP, percentage IOP reduction from baseline. Table S6. Per-eye distribution of percentage IOP reduction at 6 months in the POAG and PEX groups, by category of response magnitude. The same data are summarised in Table 5 of the main text. Median, interquartile range (IQR), and full range are also reported. IOP, intraocular pressure; IQR, interquartile range. Table S7. Post-hoc power analysis for primary within-group and between-group IOP comparisons. Within-group power was computed for the paired *t*-test; between-group power for the independent-samples *t*-test. The minimum detectable Cohen’s d at 80% power was solved for the available between-group sample size (n = 34 + 24). The translation of Cohen’s d to percentage IOP reduction units uses the average of the two groups’ standard deviations for %ΔIOP at 180 days. Figure S1. Histogram of percentage IOP reduction at 6 months in the POAG and PEX groups, illustrating the distribution of individual responses. The data underlying the figure are tabulated in Table S6 (categorical bins) and Tables S1–S2 (per-eye values). Visual emphasis is placed on the absence of any non-responder (no eye fell below the 20% reduction threshold) and the concentration of POAG responses at 30–40% reduction versus the concentration of PEX responses at 20–30% reduction. POAG, primary open-angle glaucoma; PEX, pseudoexfoliative glaucoma; IOP, intraocular pressure.
