# Supplementary material for: Outcomes of Micropulse Transscleral Cyclophotocoagulation in Primary Open-Angle and Pseudoexfoliative Glaucoma
Source: Medicina (Kaunas). 2026 May 9;62(5):920. doi: 10.3390/medicina62050920 (PMC13208441; doi:10.3390/medicina62050920)
Supplement: Supplementary file 1 [file medicina-62-00920-s001.zip › Supplementary_Material.pdf]

Supplementary Material

Outcomes of Micropulse Transscleral Cyclophotocoagulation in Primary Open-Angle and Pseudoexfoliative Glaucoma

Table S1. Individual patient-level data for the POAG group, including sex, age, baseline IOP, postoperative IOP at 30, 90, and 180 days, number of antiglaucoma medications before and after treatment, and absolute and percentage IOP reduction at each follow-up timepoint. IOP, intraocular pressure; Drops Pre-op, number of antiglaucoma medications before treatment; Drops Post-op, number of antiglaucoma medications at 6-month follow-up; ΔIOP, absolute IOP reduction from baseline; d, days; F, female; M, male.

No.	Sex	Age (yrs)	Drops Pre-op	Baseline IOP (mmHg)	IOP 30d	IOP 90d	IOP 180d	Drops Post-op	Drop change	ΔIOP 30d (mmHg)	ΔIOP 30d (%)	ΔIOP 90d (mmHg)	ΔIOP 90d (%)	ΔIOP 180d (mmHg)	ΔIOP 180d (%)
1	F	66	3	25	19	18	17	3	0	6	24.0%	7	28.0%	8	32.0%
2	F	66	3	28	20	20	19	3	0	8	28.6%	8	28.6%	9	32.1%
3	M	64	3	31	21	19	18	2	+1	10	32.3%	12	38.7%	13	41.9%
4	M	64	2	26	19	18	15	2	0	7	26.9%	8	30.8%	11	42.3%
5	M	57	3	34	24	24	23	2	+1	10	29.4%	10	29.4%	11	32.4%
6	M	57	1	22	18	17	17	1	0	4	18.2%	5	22.7%	5	22.7%
7	M	58	3	27	20	19	17	3	0	7	25.9%	8	29.6%	10	37.0%
8	M	58	3	24	19	18	18	1	+2	5	20.8%	6	25.0%	6	25.0%
9	M	67	3	26	20	18	18	3	0	6	23.1%	8	30.8%	8	30.8%
10	M	67	3	28	20	19	20	2	+1	8	28.6%	9	32.1%	8	28.6%
11	F	64	3	24	19	19	19	2	+1	5	20.8%	5	20.8%	5	20.8%
12	F	64	3	25	19	18	16	3	0	6	24.0%	7	28.0%	9	36.0%
13	M	54	3	28	22	19	19	2	+1	6	21.4%	9	32.1%	9	32.1%
14	M	54	3	24	21	20	16	2	+1	3	12.5%	4	16.7%	8	33.3%
15	F	58	3	29	24	22	22	2	+1	5	17.2%	7	24.1%	7	24.1%
16	F	58	2	25	20	18	17	2	0	5	20.0%	7	28.0%	8	32.0%
17	F	49	3	31	24	23	22	3	0	7	22.6%	8	25.8%	9	29.0%
18	F	49	3	29	23	21	21	3	0	6	20.7%	8	27.6%	8	27.6%
19	M	66	2	27	21	20	19	3	-1	6	22.2%	7	25.9%	8	29.6%
20	M	66	2	24	20	19	15	1	+1	4	16.7%	5	20.8%	9	37.5%
21	M	61	3	26	20	19	18	2	+1	6	23.1%	7	26.9%	8	30.8%
22	M	61	3	29	21	20	20	3	0	8	27.6%	9	31.0%	9	31.0%
23	M	55	3	28	21	18	18	2	+1	7	25.0%	10	35.7%	10	35.7%
24	M	55	3	30	20	17	17	3	0	10	33.3%	13	43.3%	13	43.3%
25	F	54	3	26	20	19	15	2	+1	6	23.1%	7	26.9%	11	42.3%
26	F	54	3	24	19	20	16	3	0	5	20.8%	4	16.7%	8	33.3%
27	M	66	2	23	20	20	17	2	0	3	13.0%	3	13.0%	6	26.1%
28	M	66	3	29	22	20	18	3	0	7	24.1%	9	31.0%	11	37.9%

No.	Sex	Age (yrs)	Drops Pre-op	Baseline IOP (mmHg)	IOP 30d	IOP 90d	IOP 180d	Drops Post-op	Drop change	ΔIOP 30d (mmHg)	ΔIOP 30d (%)	ΔIOP 90d (mmHg)	ΔIOP 90d (%)	ΔIOP 180d (mmHg)	ΔIOP 180d (%)
29	F	62	3	28	21	18	19	2	+1	7	25.0%	10	35.7%	9	32.1%
30	F	62	2	21	18	16	16	2	0	3	14.3%	5	23.8%	5	23.8%
31	F	63	3	21	20	15	15	2	+1	1	4.8%	6	28.6%	6	28.6%
32	F	63	2	26	20	16	18	2	0	6	23.1%	10	38.5%	8	30.8%
33	M	66	3	25	19	19	17	3	0	6	24.0%	6	24.0%	8	32.0%
34	M	66	2	28	19	18	18	2	0	9	32.1%	10	35.7%	10	35.7%

Table S2. Individual patient-level data for the PEX group, with the same column structure as Table S1. IOP, intraocular pressure; Drops Pre-op, number of antiglaucoma medications before treatment; Drops Post-op, number of antiglaucoma medications at 6-month follow-up; ΔIOP, absolute IOP reduction from baseline; d, days; F, female; M, male.

No.	Sex	Age (yrs)	Drops Pre-op	Baseline IOP (mmHg)	IOP 30d	IOP 90d	IOP 180d	Drops Post-op	Drop change	ΔIOP 30d (mmHg)	ΔIOP 30d (%)	ΔIOP 90d (mmHg)	ΔIOP 90d (%)	ΔIOP 180d (mmHg)	ΔIOP 180d (%)
1	M	58	3	28	22	19	19	3	0	6	21.4%	9	32.1%	9	32.1%
2	M	58	3	29	21	19	18	2	+1	8	27.6%	10	34.5%	11	37.9%
3	M	65	2	26	19	18	18	2	0	7	26.9%	8	30.8%	8	30.8%
4	M	65	2	25	18	17	17	2	0	7	28.0%	8	32.0%	8	32.0%
5	F	67	3	29	22	22	20	3	0	7	24.1%	7	24.1%	9	31.0%
6	F	67	3	30	24	22	22	2	+1	6	20.0%	8	26.7%	8	26.7%
7	M	64	2	24	20	19	18	2	0	4	16.7%	5	20.8%	6	25.0%
8	M	64	3	22	17	15	15	3	0	5	22.7%	7	31.8%	7	31.8%
9	F	58	2	26	18	18	17	2	0	8	30.8%	8	30.8%	9	34.6%
10	F	58	3	28	20	19	18	1	+2	8	28.6%	9	32.1%	10	35.7%
11	M	64	3	29	22	20	19	2	+1	7	24.1%	9	31.0%	10	34.5%
12	M	64	2	24	18	18	17	1	+1	6	25.0%	6	25.0%	7	29.2%
13	M	54	3	23	19	17	17	2	+1	4	17.4%	6	26.1%	6	26.1%
14	M	54	3	22	18	18	16	2	+1	4	18.2%	4	18.2%	6	27.3%
15	F	52	3	22	17	16	16	2	+1	5	22.7%	6	27.3%	6	27.3%
16	F	52	3	27	21	20	20	3	0	6	22.2%	7	25.9%	7	25.9%
17	M	56	2	26	19	20	19	2	0	7	26.9%	6	23.1%	7	26.9%
18	M	56	3	25	20	19	19	2	+1	5	20.0%	6	24.0%	6	24.0%
19	F	49	2	24	20	19	19	3	-1	4	16.7%	5	20.8%	5	20.8%
20	F	49	2	26	20	20	20	2	0	6	23.1%	6	23.1%	6	23.1%
21	M	52	3	26	19	19	18	2	+1	7	26.9%	7	26.9%	8	30.8%
22	M	52	3	24	17	16	17	1	+2	7	29.2%	8	33.3%	7	29.2%
23	F	56	2	28	22	20	20	3	-1	6	21.4%	8	28.6%	8	28.6%
24	F	56	3	29	24	24	23	2	+1	5	17.2%	5	17.2%	6	20.7%

Table S3. Full output of the linear mixed-effects model fitted to the longitudinal IOP data (n = 232 observations from 29 patient clusters). Model: IOP ~ time × group, with time as a categorical fixed effect (levels: 0, 30, 90, 180 days), group as a fixed effect (POAG as reference), and a random intercept for patient. Restricted maximum likelihood (REML) estimation; Powell optimisation. Random intercept variance = 1.697; residual scale = 2.937; intra-class correlation = 0.366.

Effect	Coefficient (β)	Standard error	z	p-value	95% CI
Intercept (POAG, baseline)	26.500	0.432	61.41	<0.001	25.65 to 27.35
Time: Day 30 vs baseline	−6.118	0.416	−14.72	<0.001	−6.93 to −5.30
Time: Day 90 vs baseline	−7.559	0.416	−18.19	<0.001	−8.37 to −6.74
Time: Day 180 vs baseline	−8.559	0.416	−20.59	<0.001	−9.37 to −7.74
Group (PEX vs POAG, at baseline)	−0.583	0.671	−0.87	0.385	−1.90 to +0.73
Time × Group: Day 30 × PEX	+0.076	0.646	+0.12	0.906	−1.19 to +1.34
Time × Group: Day 90 × PEX	+0.559	0.646	+0.87	0.387	−0.71 to +1.83
Time × Group: Day 180 × PEX	+1.059	0.646	+1.64	0.101	−0.21 to +2.33
Random intercept variance (between patients)	1.697	—	—	—	—
Residual variance (within patients)	2.937	—	—	—	—

Table S4. Full output of the GEE analysis of the longitudinal IOP data (n = 232 observations from 29 patient clusters). Model specification: IOP ~ time × group; identity link, Gaussian family, exchangeable working correlation, robust (sandwich) standard errors. Estimated working correlation parameter = 0.346. Cluster = patient.

Effect	Coefficient (β)	Robust SE	z	p-value	95% CI
Intercept (POAG, baseline)	26.500	0.408	65.02	<0.001	25.70 to 27.30
Time: Day 30 vs baseline	−6.118	0.332	−18.45	<0.001	−6.77 to −5.47
Time: Day 90 vs baseline	−7.559	0.355	−21.29	<0.001	−8.26 to −6.86
Time: Day 180 vs baseline	−8.559	0.327	−26.16	<0.001	−9.20 to −7.92
Group (PEX vs POAG)	−0.583	0.726	−0.80	0.421	−2.01 to +0.84
Time × Group: Day 30 × PEX	+0.076	0.464	+0.16	0.870	−0.83 to +0.99
Time × Group: Day 90 × PEX	+0.559	0.508	+1.10	0.271	−0.44 to +1.55
Time × Group: Day 180 × PEX	+1.059	0.510	+2.08	0.038	+0.06 to +2.06

Table S5. Sensitivity analysis using 1000 random subsamples drawn from the dataset, each containing one randomly selected eye per patient. Reports the mean of each estimate across all 1000 iterations, the 2.5–97.5 percentile range, and the proportion of iterations in which the relevant statistical test reached $p < 0.05$. The proportion of significant iterations reflects the robustness of the primary findings to the inclusion of one eye versus the other from bilateral patients. POAG, primary open-angle glaucoma; PEX, pseudoexfoliative glaucoma; %ΔIOP, percentage IOP reduction from baseline.

Estimate	POAG (mean across iterations; 2.5–97.5 percentile)	PEX (mean across iterations; 2.5–97.5 percentile)
Mean IOP at 180 days (mmHg)	17.95 (17.47–18.41)	18.42 (18.33–18.50)
Mean %ΔIOP at 180 days	32.07% (31.62–32.53)	28.83% (28.62–29.04)
Paired test baseline vs 180d significant ($p < 0.05$): % of iterations	100.0%	100.0%
Success rate (≥20% IOP reduction)	100.0%	100.0%
Success rate (Tekeli criterion A)	67.5%	54.1%
Success rate (Tekeli criterion B)	11.8%	4.1%

Additional sensitivity analysis statistics: Between-group test of %ΔIOP at 180 days reached $p < 0.05$ in 0.0% of iterations (i.e., zero of 1000 subsamples), and the median Cohen's d for the between-group comparison was +0.516. This indicates that the borderline between-group difference observed in the unadjusted main-text analysis is sensitive to the specific eye selected for bilateral patients and is not robust to the one-eye-per-patient analytic strategy.

Table S6. Per-eye distribution of percentage IOP reduction at 6 months in the POAG and PEX groups, by category of response magnitude. The same data are summarised in Table 5 of the main text. Median, interquartile range (IQR), and full range are also reported. IOP, intraocular pressure; IQR, interquartile range.

Response category at 180 days	POAG (n = 34)	PEX (n = 24)
IOP increase or no change	0/34 (0.0%)	0/24 (0.0%)
0 to <10% reduction	0/34 (0.0%)	0/24 (0.0%)
10 to <20% reduction	0/34 (0.0%)	0/24 (0.0%)
20 to <30% reduction	11/34 (32.4%)	14/24 (58.3%)
30 to <40% reduction	19/34 (55.9%)	10/24 (41.7%)
≥40% reduction	4/34 (11.8%)	0/24 (0.0%)
Median %ΔIOP at 180 days	32.0%	28.9%
IQR	28.7–35.7%	26.1–31.9%
Range	20.8–43.3%	20.7–37.9%

Table S7. Post-hoc power analysis for primary within-group and between-group IOP comparisons. Within-group power was computed for the paired t-test; between-group power for the independent-samples t-test. The minimum detectable Cohen's d at 80% power was solved for the available between-group sample size (n = 34 + 24). The translation of Cohen's d to percentage IOP reduction units uses the average of the two groups' standard deviations for %ΔIOP at 180 days.

Comparison	Sample size	Observed effect size	Achieved power
POAG: paired test, baseline vs 180d	34 eyes	Cohen's d = 4.24	1.00 (>0.99)
PEX: paired test, baseline vs 180d	24 eyes	Cohen's d = 4.81	1.00
Between-group: %ΔIOP at 180d (POAG vs PEX)	34 + 24 eyes	Cohen's d = 0.57	0.56
Detectable effect size at 80% power, between-group	34 + 24 eyes	—	Cohen's d = 0.76 (≈ 3.9% Δ in %ΔIOP)

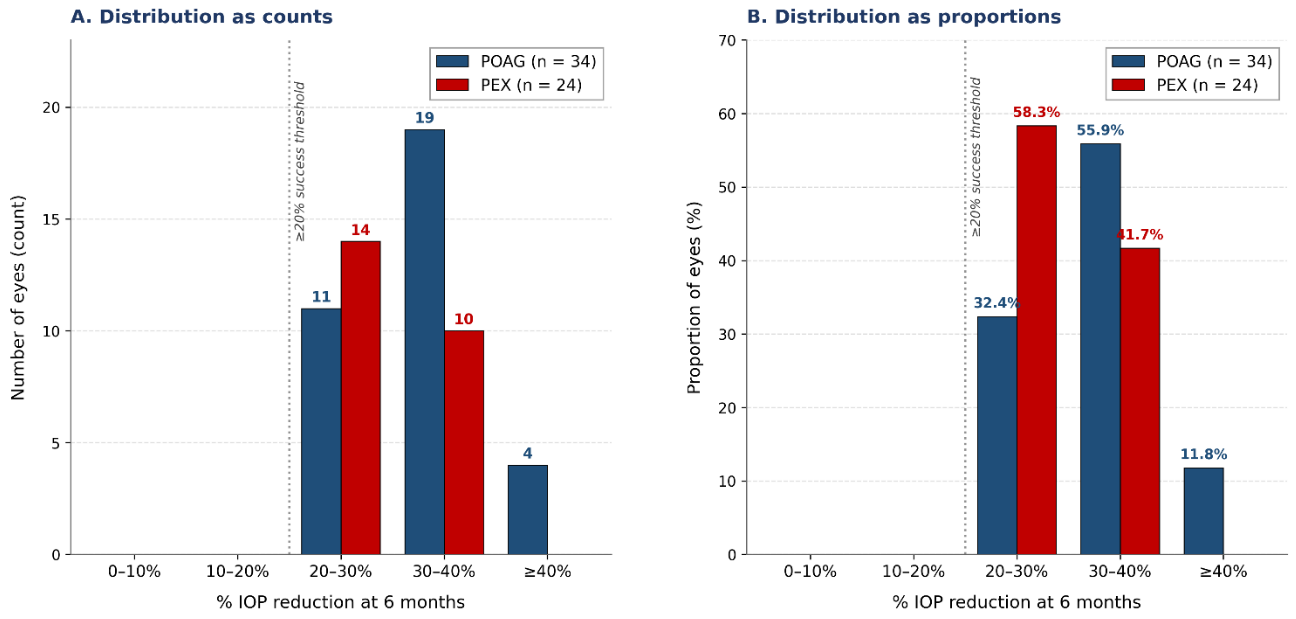

Median % IOP reduction at 180 days: POAG 32.0% (IQR 28.7–35.7; range 20.8–43.3); PEX 28.9% (IQR 26.1–31.9; range 20.7–37.9). No eye in either group experienced IOP increase or reduction below the 20% success threshold.

Figure S1. Histogram of percentage IOP reduction at 6 months in the POAG and PEX groups, illustrating the distribution of individual responses. The data underlying the figure are tabulated in Table S6 (categorical bins) and Tables S1–S2 (per-eye values). Visual emphasis is placed on the absence of any non-responder (no eye fell below the 20% reduction threshold) and the concentration of POAG responses at 30–40% reduction versus the concentration of PEX responses at 20–30% reduction. POAG, primary open-angle glaucoma; PEX, pseudoexfoliative glaucoma; IOP, intraocular pressure.

Note S1. Operational definitions used in the analysis

This note summarises the operational definitions of statistical procedures and outcome criteria used in the main analysis, for reference in interpreting the supplementary tables.

Patient clustering for mixed-effects and GEE analyses. In the absence of de-identified patient-level identifiers in the operational data file, patient identity for bilateral cases was inferred by pairing adjacent rows in the original individual-data tables when sex and age were identical. This conservative pairing yielded 29 patient clusters (17 POAG + 12 PEX). This approach overestimates rather than underestimates intra-cluster correlation, providing the most stringent test of cluster-adjusted findings; if results are robust under this assumption, they will be at least as robust under the true cluster structure.

Cohen's d for paired data. Computed as the mean difference between paired observations divided by the standard deviation of the differences.

Cohen's d for independent samples. Computed as the difference in group means divided by the pooled standard deviation, where the pooled SD uses an n−1 weighting per group.

Wilson score 95% confidence interval for proportions. Used in preference to the normal-approximation interval because it provides accurate coverage for small samples and proportions near 0 or 1.

TOST procedure for equivalence testing. Two one-sided t-tests are performed against the lower (−margin) and upper (+margin) bounds of the equivalence margin. Equivalence is concluded if both one-sided tests are rejected at $\alpha = 0.05$, equivalent to checking that the 90% CI of the difference lies entirely within the equivalence interval.

Tekeli & Köse composite success criteria [22]. Three tiered criteria of increasing stringency: criterion A requires IOP ≤ 18 mmHg AND ≥2 0% reduction; criterion B requires IOP ≤ 15 mmHg AND ≥ 25% reduction; criterion C requires IOP ≤ 12 mmHg AND ≥ 30% reduction.

Random one-eye-per-patient sensitivity analysis. For each of 1000 iterations: for each patient with multiple eyes, one eye is randomly selected (uniform probability); for unilateral patients, the single eye is retained; the resulting one-eye-per-patient subsample (n = 29 eyes) is subjected to the primary statistical tests; the test results are stored. Across all 1000 iterations, the mean and 2.5–97.5 percentile of each estimate are reported, together with the proportion of iterations in which the test reached statistical significance at $p < 0.05$.
