# Supplementary figures and images for: Outcomes of Micropulse Transscleral Cyclophotocoagulation in Primary Open-Angle and Pseudoexfoliative Glaucoma
Source: Medicina (Kaunas). 2026 May 9;62(5):920. doi: 10.3390/medicina62050920 (PMC13208441; doi:10.3390/medicina62050920)

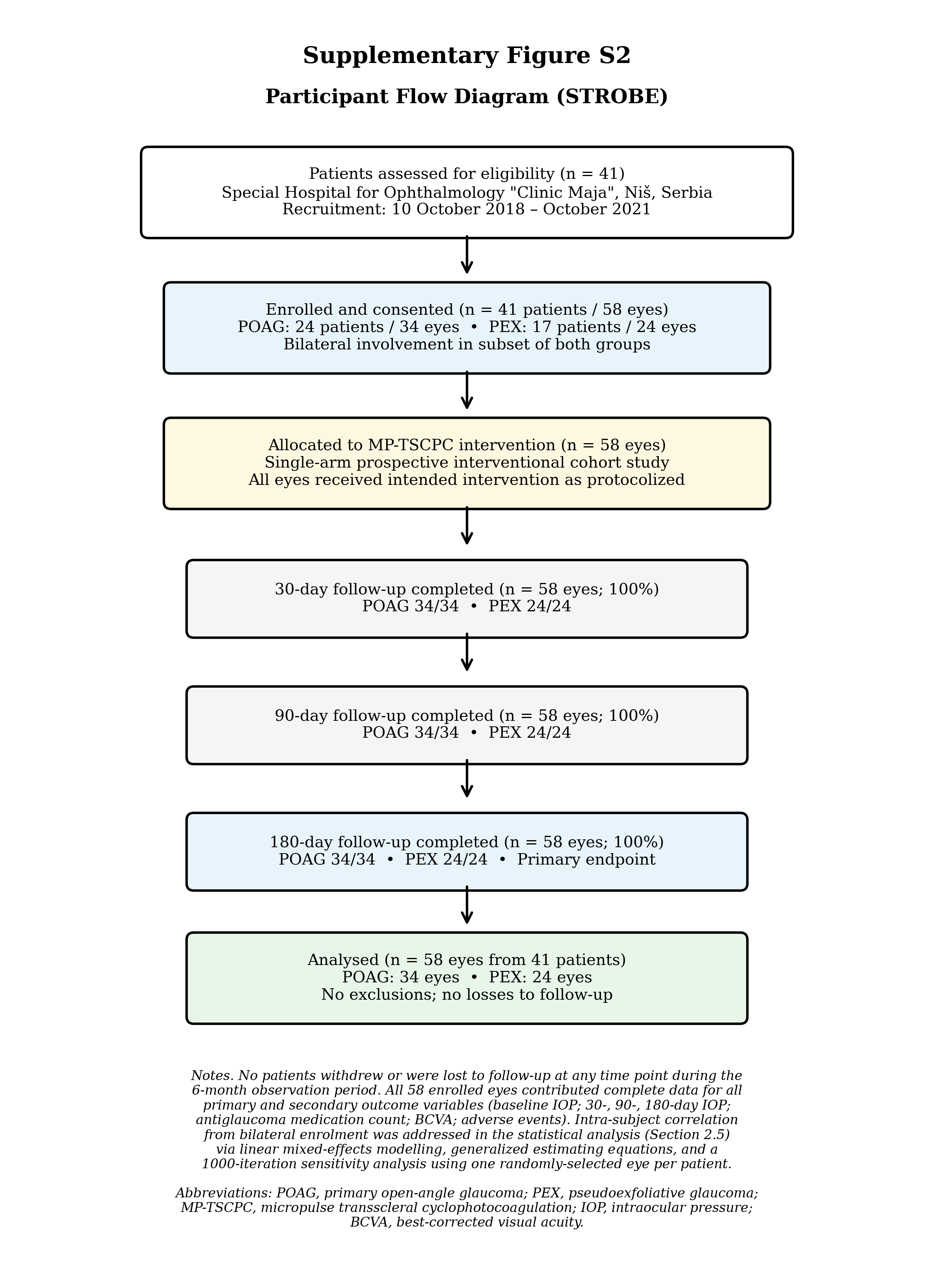

Supplement: Supplementary file 1 [file medicina-62-00920-s001.zip › FigureS2_participant_flow.png]
